# The ncRNA-AURKA Interaction in Hepatocellular Carcinoma: Insights into Oncogenic Pathways, Therapeutic Opportunities, and Future Challenges

**DOI:** 10.3390/life14111430

**Published:** 2024-11-06

**Authors:** Clarissa Joy C. Garcia, Luca Grisetti, Claudio Tiribelli, Devis Pascut

**Affiliations:** 1 Liver Cancer Unit, Fondazione Italiana Fegato—ONLUS, 34149 Trieste, Italy; 2 Department of Life Sciences, Università degli Studi di Trieste, 34127 Trieste, Italy; 3 National Institute of Gastroenterology—IRCCS “Saverio de Bellis”, 70013 Castellana Grotte, Italy

**Keywords:** Aurora kinase A, microRNA, long-non-coding RNA, circular RNA, hepatocellular carcinoma

## Abstract

Hepatocellular carcinoma (HCC) represents a major public health concern and ranks among the leading cancer-related mortalities globally. Due to the frequent late-stage diagnosis of HCC, therapeutic options remain limited. Emerging evidence highlights the critical role of non-coding RNAs (ncRNAs) in the regulation of Aurora kinase A (AURKA), one of the key hub genes involved in several key cancer pathways. Indeed, the dysregulated interaction between ncRNAs and AURKA contributes to tumor development, progression, and therapeutic resistance. This review delves into the interplay between ncRNAs and AURKA and their role in hepatocarcinogenesis. Recent findings underscore the involvement of the ncRNAs and AURKA axis in tumor development and progression. Furthermore, this review also discusses the clinical significance of targeting ncRNA-AURKA axes, offering new perspectives that could lead to innovative therapeutic strategies aimed at improving outcomes for HCC patients.

## 1. Introduction

Hepatocellular carcinoma (HCC) accounts for approximately 90% of all liver cancer cases and is the third leading cause of cancer-related deaths globally, with an estimated death toll of 758,725 in 2022 [1,2,3]. The incidence rates of HCC vary by region due to differences in risk factor prevalence [4]. Regions with high incidence rates of hepatitis B and C infections, such as East Asia and sub-Saharan Africa, experience the highest burden of HCC [3,5]. Recent predictions suggest that the global burden of HCC will continue to rise, especially in developing countries where access to vaccines and anti-viral treatments is limited [2]. HCC is often diagnosed at late stages when the treatment options are limited and only effective for a small subset of patients [6]. Hence, HCC is not only a disease that negatively impacts quality of life but also bears a substantial economic burden, with costs stemming from hospitalizations, liver transplants, and treatment [7]. In countries with limited healthcare resources, this economic impact is exacerbated by late-stage diagnosis and the need for expensive targeted drugs and immunotherapies. The cost-effectiveness of these treatments remains a challenge, and access to care is a significant public health concern, particularly in developing nations [8].

HCC development is influenced by a combination of genetic and environmental factors. Chronic infections with hepatitis B virus (HBV) and hepatitis C virus (HCV) remain the leading causes of HCC, accounting for over 80% of cases globally [9]. Other relevant risk factors include metabolic-dysfunction-associated steatotic liver disease (MASLD) [10], alcohol abuse [11], and aflatoxin exposure [12], with metabolic diseases such as obesity and type 2 diabetes becoming increasingly significant in the Western world [13]. At the molecular level, HCC shows a heterogeneous profile of genetic mutations, epigenetic alterations, and copy number variations (CNV) in key driver genes, leading to the disruption of critical signaling pathways [14]. However, recurring alterations exist, such as telomerase reactivation, which occurs in 80% of HCC cases, and the activation of the Wingless-related integration site (Wnt)–catenin beta 1 (CTNNB1) signaling pathway, observed in 30–50% of cases. Other crucial alterations involve genes responsible for cell cycle regulation, such as *Tumor protein P53* (*TP53*) and *Cyclin-dependent kinase inhibitor 2A* (*CDKN2A*) [1]. Additionally, *Aurora kinase A (AURKA)* is frequently overexpressed in HCC, where it plays a pivotal role in promoting cell survival [15,16], proliferation [17,18], migration, and invasion [19,20]. The overexpression of *AURKA* results from gene amplification, single-point mutations (SNPs), and modulation by non-coding RNAs (ncRNAs) [21,22,23]. Moreover, AURKA can modulate the expression of ncRNAs, further contributing to disease progression [24].

NcRNAs can act either as tumor suppressors or oncogenes by interacting with DNA, RNA, or proteins [25,26,27,28]. Aberrant ncRNA expression contributes to hepatocarcinogenesis by affecting cancer stem cell (CSC) formation, tumor immune microenvironment, immune cell infiltration, angiogenesis, metabolism, epithelial-mesenchymal transition (EMT), invasion, metastasis, and drug resistance [29,30,31].

This review investigates the intricate interplay between AURKA and ncRNAs in HCC, focusing on how ncRNAs regulate AURKA expression and activity and how AURKA modulates ncRNAs. Understanding these interactions could pave the way for identifying novel biomarkers and therapeutic strategies in HCC management.

## 2. The Roles of AURKA

**Figure 1:**
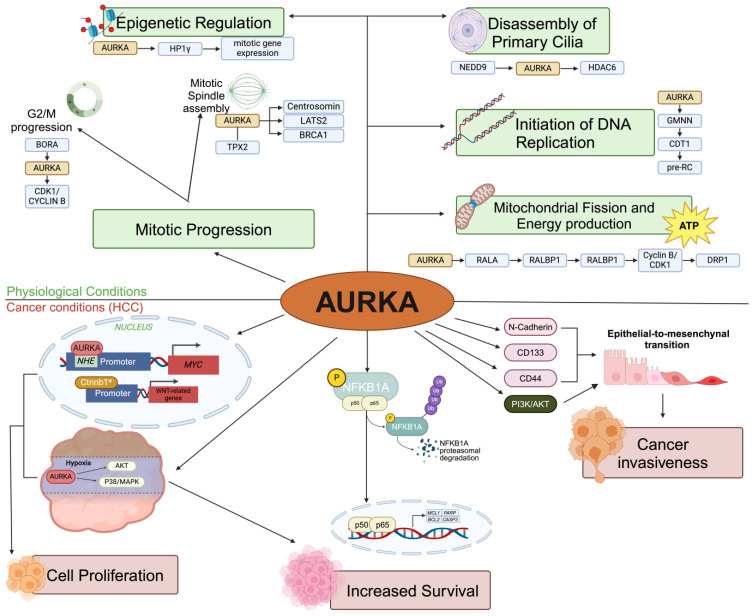
**The multifaceted roles of AURKA.** Under physiological conditions (green), AURKA plays a key function in mitotic progression, including G2/M checkpoint release, mitotic spindle formation, organization, and epigenetic regulation. It also contributes to the disassembly of primary cilia, initiation of DNA replication, and regulation of mitochondrial fission and energy production. AURKA interacts with numerous proteins and participates in diverse signaling pathways; thus, its overexpression in cancer leads to the dysregulation of these pathways, driving oncogenic effects. In the context of cancer (red), AURKA enhances cell survival and proliferation, epithelial-mesenchymal transition (EMT), and cancer invasiveness.

AURKA is a serine/threonine kinase belonging to the Aurora family, pivotal in orchestrating various cellular processes (Figure 1). Its kinase activity is mainly regulated by the phosphorylation of the Thr288 residue, a post-translational modification essential for its function [32,33].

### 2.1. The Functions of AURKA Under Physiological Conditions

In physiological contexts, AURKA is critical for regulating mitosis [34]. It is involved in the G2/M transition, centrosome maturation, mitotic entry, microtubule organization, spindle assembly, and cytokinesis [35,36] (Figure 1). At the end of the G2 phase, the activation of AURKA is mediated by phosphorylation through BORA Aurora kinase A activator (BORA). This event triggers the Cyclin-dependent kinase 1 (CDK1)–Cyclin B complex, which, in turn, unlocks the G2/M checkpoint, allowing the cell to progress into mitosis (Figure 1). Once activated, AURKA localizes to the centrosome, where it phosphorylates several pericentriolar material proteins, such as Centrosomin, Large tumor suppressor 2 (LATS2), and Breast cancer type 1 (BRCA1), promoting microtubule nucleation, a fundamental step in mitotic spindle formation (Figure 1). During metaphase, AURKA organizes the mitotic spindle by interacting with TPX2 microtubule nucleation factor (TPX2) [37] (Figure 1). Beyond its structural functions, AURKA also plays a pivotal role in epigenetic regulation during the G2/M phase. AURKA specifically phosphorylates Heterochromatin Protein 1 gamma (HP1γ) on Ser83, a key reader of histone H3K9 methylation. This modification affects the mitotic gene expression network, thereby facilitating cell cycle progression and promoting cell proliferation [38] (Figure 1). Mutations in AURKA result in abnormal centrosome duplication and separation, leading to defective spindle formation and consequent incorrect chromosome separation [39,40].

AURKA is involved in several cellular processes beyond its well-established role in mitosis [41]. During interphase, AURKA autophosphorylation on Thr288 leads to the disassembly of primary cilia, a process associated with cell cycle re-entry [42] (Figure 1). This disassembly is driven by AURKA’s interaction with Neural precursor cells expressed developmentally downregulated 9 (NEDD9), which promotes its autophosphorylation and subsequently increases the activity of Histone deacetylase 6 (HDAC6), contributing to primary cilia disassembly [43] (Figure 1). Moreover, AURKA plays a role in regulating the initiation of DNA replication. During the M phase, AURKA stabilizes Geminin (GMNN) through phosphorylation of the Thr25 residue, thereby protecting Chromatin licensing and DNA replication factor 1 (CDT1) from degradation and promoting the proper functioning of the pre-replicative complex (pre-RC) [44,45] (Figure 1).

AURKA also plays a role in the mitochondria, where it regulates morphology and energy production through the phosphorylation of Dynamin-Related Protein 1 (DRP1) on Ser616 [46] (Figure 1). Specifically, AURKA phosphorylates RAS-like proto-oncogene A (RALA) on Ser194, promoting the recruitment of RalA binding protein 1 (RALBP1). Additionally, RALBP1 binds to the Cyclin B/CDK1 complex, which induces the phosphorylation of DRP1 on Ser616, thus promoting mitosis-induced mitochondrial fission [47] (Figure 1).

### 2.2. The Functions of AURKA in Hepatocellular Carcinoma

AURKA overexpression in HCC is closely linked with cancer progression, influencing several key cellular pathways responsible for tumor proliferation, survival, migration, and invasion. AURKA regulates phosphatidylinositol-3-kinase (PI3K)/protein kinase B (AKT) and Catenin Beta 1 (CTNNB1), which drive malignant transformation [21,22,23] (Figure 1). AURKA is also known to promote uncontrolled cellular proliferation, a hallmark of cancer. Its nuclear localization enables it to regulate oncogenes like MYC, enhancing its transcriptional activity, which leads to aggressive cancer behavior. Studies in HepG2 and BEL-7402 cells revealed that AURKA binds to MYC’s nuclear hypersensitive element, elevating its expression, with positive consequences in terms of cell proliferation, metastatic ability, and drug resistance [48] (Figure 1). Under hypoxic conditions, AURKA can promote cancer cell survival and proliferation *via* the AKT and p38/mitogen-activated protein kinase (MAPK) cascades (Figure 1). Indeed, AURKA inhibition reduces AKT and p38 signaling, thereby attenuating these effects [17]. Additionally, in a zebrafish HCC model, mutant AURKA overexpression led to reduced membrane-bound Ctnnb1, thus allowing for the nuclear translocation of Ctnnb1, highlighting its involvement in the Wnt/CTNNB1 signaling pathway [49] (Figure 1). AURKA upregulation following radiation in HCC cells is associated with EMT, a process that enhances metastatic potential and drug resistance in HCC. Increased expression of EMT markers (N-cadherin) and CSC markers (CD133, CD44) has been observed, contributing to the chemoresistance often seen in HCC [19,50] (Figure 1). By regulating the PI3K/AKT and MAPK pathways, AURKA enhances cell invasiveness and EMT in HCC cells [19,22] (Figure 1).

AURKA also plays a role in resistance to apoptosis, a key challenge in cancer treatment. It activates the nuclear factor kappa B (NF-κB) signaling pathway by phosphorylating NF-κB inhibitor alpha (NFKBIA), leading to the ubiquitin-mediated proteasomal degradation of NFKBIA and subsequent nuclear translocation of the NF-κB complex (Figure 1). This promotes the expression of anti-apoptotic genes like MCL1 Apoptosis Regulator, BCL2 family member (MCL1), BCL2 apoptosis regulator (BCL2), Poly ADP-ribose polymerase (PARP), and Caspase 3 (CASP3), contributing to cell survival [51,52] (Figure 1). Moreover, AURKA regulates the expression of miR-21, which inhibits Phosphatase and tensin homolog deleted on chromosome 10 (PTEN) and blocks CASP3-mediated apoptosis by upregulating anti-apoptotic proteins such as p-AKT and BCL2. This signaling axis (AURKA/NF-κB/miR-21/PTEN) is linked to chemoresistance in HCC, further complicating treatment strategies [24]. 

## 3. Regulatory ncRNAs: Types and Functions

Regulatory non-coding RNAs (ncRNAs) have emerged as pivotal players in the complex landscape of gene regulation and cellular processes, despite their lack of protein-coding capacity. Initially dismissed as mere “junk” DNA owing to their origins in repetitive and transposable elements within the human genome, ncRNAs have become a crucial area of research, especially in cancer [53]. In contrast to housekeeping ncRNAs, regulatory ncRNAs exhibit a more restricted expression pattern, often demonstrating tissue- and developmental stage-specificity. This specificity enhances their potential clinical utility in cancer and therapy. Several classes of ncRNAs exist, each featuring distinct structures and functions, adding to their diverse regulatory roles in cellular processes [54]. Among them are microRNAs (miRNAs), long non-coding RNAs (lncRNAs), and circular RNAs (circRNAs), for which the biogenesis and mechanisms of action (MoA) have been extensively reviewed by others [55,56,57]. Recent technological strides, particularly in transcriptomics and RNA sequencing, have paved the way for more advanced studies of ncRNAs in cancer [58,59,60,61]. Thus, understanding their roles not only unveils fundamental cellular mechanisms but also sheds light on their significance in cancer biology.

### ncRNAs: miRNAs, lncRNAs, and circRNAs

**Figure 2:**
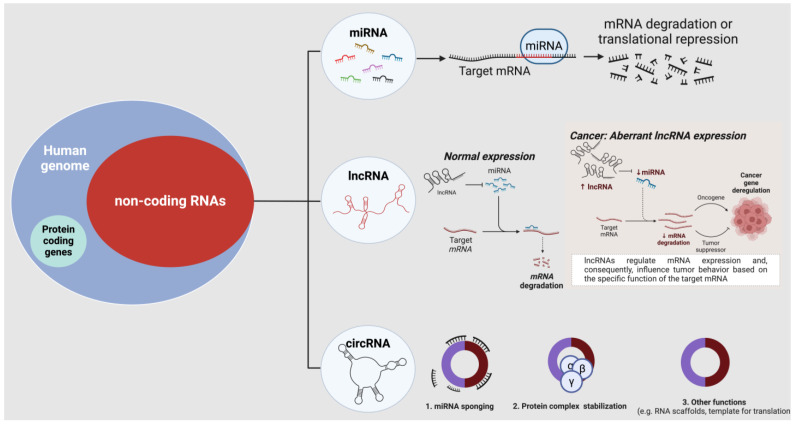
**Regulatory ncRNAs: From physiology to pathology.** Regulatory ncRNAs are a fraction of the ncRNAs within the human genome. MicroRNAs (miRNAs), long-non-coding RNAs (lncRNAs), and circular RNAs (circRNAs) are the major players involved in regulating gene expression and protein levels and stability in physiological and pathological conditions.

The most extensively investigated class of ncRNAs is represented by miRNAs, characterized by their small size of around 22 nucleotides and high evolutionary conservation [62]. They play a crucial role in post-transcriptional regulation, which accounts for approximately 60% of regulation in all human protein-coding genes [63]. This regulation occurs through the binding of miRNAs to complementary sequences within the 3′ untranslated region (3′-UTR) of target messenger RNAs (mRNAs), resulting in translational repression or mRNA degradation [64,65,66] (Figure 2). Dysregulation of miRNAs in cancer is a frequently observed phenomenon and has been mechanistically linked to chromosomal defects [67], transcriptional control changes [68], epigenetic aberrations [69], and miRNA biogenesis defects [70]. Furthermore, high-throughput profiling of differentially expressed genes has facilitated the identification and functional characterization of miRNAs that play a key role in several oncogenic processes, with clinical data validating their potential as biomarkers in various cancer types [71].

LncRNAs constitute a diverse group of transcripts that exceed 200 nucleotides in length and are now widely recognized as crucial regulators of transcriptional and post-transcriptional processes, employing various mechanisms to modulate gene expression, largely depending on their sub-cellular localization [72,73] (Figure 2). Despite their diversity, lncRNAs share common features such as low sequence conservation and tissue-specificity, hence their differential expression patterns in tumors. The dysregulated expression of lncRNAs is linked to increased transforming growth capacity, invasiveness and metastatic ability of cancer cells, and poor patient outcome in several cancers [74,75,76,77,78] (Figure 2).

circRNAs represent a relatively novel class of regulatory ncRNAs characterized by their covalently closed loop structures, which confer resistance to exonucleases, resulting in enhanced stability compared to linear RNAs [27]. In cancer, circRNAs act as important regulators of tumorigenesis and progression. Their ability to sponge miRNAs, thereby regulating the expression of oncogenes and tumor suppressors, contributes to proliferation, invasion, and metastasis [79,80]. Additionally, circRNAs can function as scaffolds for proteins involved in oncogenic pathways and may also enhance protein functions by inhibiting key tumor suppressors [81,82]. Recent information has also shed light on their potential as templates for translation [82,83] (Figure 2). The dysregulation of circRNAs in many types of cancers underscores their significance as diagnostic and prognostic indicators. Thus, understanding the role of circRNAs in cancer holds promise in developing novel diagnostic tools and targeted therapies.

## 4. Interactions Between ncRNAs and AURKA in HCC

### 4.1. miRNAs Regulating AURKA in HCC

**Figure 3:**
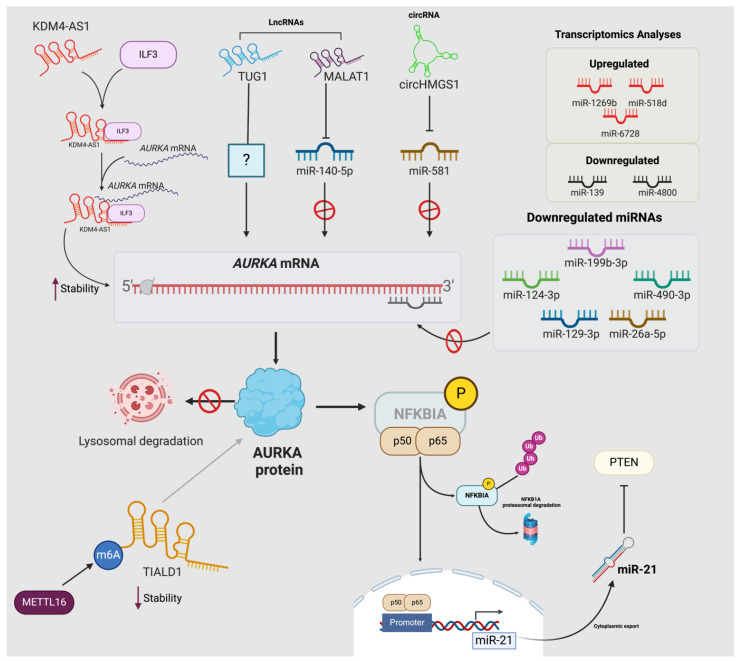
**The regulatory network of AURKA and ncRNAs in HCC**. Regulatory ncRNAs influence the expression of AURKA both at the mRNA and protein levels in HCC. MiRNAs targeting AURKA, such as miR-199-3p [96], miR-124-3p [92], miR-490-3p [89], miR-129-3p [99], and miR-26a-5p [101], are frequently downregulated in HCC. Transcriptomic analyses revealed upregulation of miR-1269b, miR-518d, and miR-6728 and downregulation of miR-139 and miR-4800 [95]. AURKA indirectly upregulates miR-21 expression by activating NF-κB signaling. The p50/p65 complex binds to the miR-21 promoter sequence, thus promoting its transcription. In turn, miR-21 represses PTEN, the negative regulator of the PI3K/AKT pathway [24]. LncRNAs such as *MALAT1* [104] and *TUG1* [105] and the circRNA circHMGS1 [106] positively regulate AURKA expression, while *KDM4A-AS1* forms a complex with ILF3 to recruit and stabilize AURKA [20]. M6A-mediated hypermethylation of *TIALD1* effectively blocks AURKA protein from lysosomal degradation [107].

miRNAs are indispensable post-transcriptional regulators during carcinogenesis [28,84,85], significantly impacting HCC development and progression [86,87,88]. *AURKA*, due to its role in regulating multiple pathways, stands out as one of the key targets of several miRNAs in HCC (Figure 3).

For instance, miR-490-3p suppressed proliferation, migration, and invasion of HepG2 and Hep3B cells by targeting *AURKA* [89] (Figure 3 and Table 1). Moreover, the downregulation of these miRNAs in HCC tissues and cell lines, coupled with their correlation with poor clinical outcomes, solidifies their function as tumor-suppressor miRNAs [90]. By analyzing two gene microarray datasets (GSE89377 and GSE101685) and two miRNA expression profiles datasets, Wang et al. identified miR-124-3p and *AURKA* as important regulators in the disease (Figure 3 and Table 1) [91]. Interestingly, miR-124-3p has emerged as a key tumor-suppressor miRNA in HCC. Overexpression of miR-124-3p *in vitro* (HCCLM3 and Huh7 cell lines) significantly inhibited proliferation, migration, and invasion by binding to the 3′-UTR of V-crk sarcoma virus CT10 oncogene homolog (avian)-like (*CRKL)* [92]. Although direct evidence of how miR-124-3p regulates *AURKA* remains unknown in HCC, existing literature supports the targeting of *AURKA* in bladder cancer [93] and glioblastoma [94]. This underscores the need for further investigation into its functional interactions and potential therapeutic implications in HCC.

Transcriptomic analyses conducted by Ma and colleagues, utilizing four datasets (GSE36376, GSE39791, GSE57957, and GSE87630), confirmed the upregulation of *AURKA* in HCC and its association with poor patient outcomes. The study identified the upregulation of miR-1269b, miR-518d, and miR-6728, and the downregulation of miR-139 and miR-4800 in HCC (Figure 3 and Table 1). These miRNAs have been proposed as poor prognostic indicators and were predicted to target *AURKA*. However, experimental evidence supporting these mechanistic interactions is currently lacking [95].

On the other hand, miR-199b-3p presents a more direct evidence by negatively regulating *AURKA in vitro* (HepG2 and SK-HEP1) and *in vivo* (BALB/c nude mice) (Figure 3 and Table 1). The overexpression of miR-199b-3p downregulated AURKA expression, subsequently inhibiting the proliferation, migration, and invasion of HCC cells while concurrently promoting apoptosis *in vitro* [96]. These results were further validated *in vivo*, where the enforced expression of miR-199b-3p in mice reduced tumor growth. However, the concomitant overexpression of AURKA counteracted these effects, suggesting a direct regulatory relationship between miR-199b-3p and *AURKA* in HCC progression [96]. The miR-199b-3p/AURKA axis was further associated with the regulation of the PI3K/AKT pathway, a well-established oncogenic signaling pathway implicated in HCC metastasis. Specifically, miR-199b-3p overexpression resulted in decreased levels of p-AKT, thus suggesting an important role of AURKA in the activation of PI3K/AKT pathway in HCC (Figure 3 and Table 1) [96].

miR-129-3p is also an important tumor suppressor miRNA in HCC, with established implications in metastasis across various cancers [97,98] (Figure 3). In this context, Cui and colleagues observed significantly higher *AURKA* expression and lower miR-129-3p expression in HCC tissues (n=88) with lymph node metastasis (LNM) and vascular invasion, among other poor prognostic indicators in their cohort. In *in vitro* models of liver cancer cell lines, the downregulation of miR-129-3p was associated with a reduced expression of epithelial markers (Cadherin 1 [CDH1] and CTNNB1) and an increase in mesenchymal markers (Cadherin 2 [CDH2] and Vimentin [VIM]) [99]). Further studies proved that the downregulation of miR-129-3p in HCC was attributed to promoter hypermethylation, which prevented miR-129-3p from directly targeting and repressing *AURKA* [99]. Consequently, AURKA can phosphorylate AKT and P38-MAPK, thus activating the PI3K/AKT and P38-MAPK pathways to promote EMT. Suppression of AURKA either by short hairpin RNA (shRNA) or miR-129-3p upregulation in HepG2 and BEL-7402 cells impaired EMT and reduced the *in vitro* migration and invasion of HCCLM3 and MHCC97-H *via* wound healing assay. Similarly, *in vivo*, metastasis was reduced following miR-129-3p upregulation in female BALB/c athymic nude mice [99] (Figure 3 and Table 1).

Chemotherapeutic resistance remains an unmet clinical need in HCC management and typically contributes to the progression of the disease [100]. miR-26a-5p was demonstrated to directly target *AURKA* in HCC cells, with a significantly lower expression observed in HCC tissues and cell lines (Figure 3). *In vitro*, the overexpression of miR-26a-5p significantly inhibited proliferation and increased the sensitivity of HCC cells (HuH7 and SMMC-7721) to doxorubicin 48h post-treatment [101]. Similarly, miR-26a-5p was also downregulated in hepatoblastoma tissues, with drastically reduced levels in stage four of the disease. Its overexpression decreased proliferation and foci formation *in vitro* (Hep293TT, HuH6, and HepG2) (Figure 3 and Table 1). Given its consistent downregulation and functional effects in both hepatoblastoma and HCC, miR-26a-5p emerges as a promising therapeutic candidate for various forms of liver cancer [102].

Since several lines of evidence have described *AURKA* as a hub gene in HCC, with key roles in carcinogenesis, understanding its regulation by miRNAs such as miR-129-3p and miR-26a-5p is crucial. These miRNAs not only modulate *AURKA* expression but also influence key pathways involved in tumor progression and metastasis. The consistent downregulation of these miRNAs in HCC and their significant impact on cell proliferation, migration, and treatment sensitivity highlight their relevance in the disease.

### 4.2. AURKA Participates in the Regulation of miRNAs in HCC

The emerging role of AURKA as a hub gene in HCC underscores its significance as a fundamental kinase and a potential therapeutic target. Its functions extend beyond the phosphorylation of direct substrates, integrating into a complex regulatory network that involves ncRNAs at various levels. Particularly, AURKA can modulate miRNA expression, contributing to the malignant phenotype.

The study of Zhang et al. demonstrated the miRNA-mediated oncogenic effects of AURKA in HCC cells (HepG2, Hep3B, and SMMC-7721). Specifically, increased AURKA promotes NF-κB signaling, which mediates the transcription of miR-21, a PTEN repressor (Figure 3). This exacerbates AKT signaling, effectively reducing not only Caspase-3-mediated apoptosis but also chemotherapy-induced apoptosis (*via* doxorubicin/adriamycin and cisplatin) in HCC cells [24]. Hence, the overexpression of AURKA promoted chemoresistance in HCC through the NF-κB/miR-21/PTEN/AKT signaling axis, underscoring the potential of AURKA in regulating multiple signaling pathways.

Despite the limited evidence about the possible role of AURKA in regulating miRNAs in HCC, other cancers may suggest the existence of new AURKA-miRNA regulatory axes not yet explored in liver cancers. This could be the case of the miR17-92 cluster induced by AURKA, through E2F transcription factor 1 (E2F1) in breast cancer [103].

**Table 1:** Non-coding regulatory network of AURKA in HCC.

ncRNAs	Experimental Model	Mechanism of Action	Effect (s)	Effects on AURKA Expression	Expression in HCC
miR-199b-3p [96]	HepG2, SK-HEP1; BALB/c nude mice (xenograft)	3′-UTR binding	Decreased proliferation, migration, and invasion. Increased apoptosis; Reduced tumor growth	Down	Down
miR-129-3p [99]	HCCLM3, MHCC97-H, HepG2, BEL-7402, normal hepatocytes (HH), BALB/c mice	3′-UTR binding	Reduced activation of the PI3K/AKT and P38/MAPK pathways, reducing EMT	Down	Down
miR-490-3p [89]	HepG2, Hep3B,	3′-UTR binding	Decreased proliferation, migration, and invasion	Down	Down
miR-26a-5p [101]	HCC: HuH-7, HCC-LM3, HepG2, Hep3B, SMMC-7721 Hepatoblastoma: Hep293T, HuH6m, HepG2	3′-UTR binding	Decreased proliferation, increased sensitivity to doxorubicin Reduced proliferation and foci formation	Down	Down
miR-21 [24]	HepG2, Hep3B, SMMC-7721	AURKA increased the transcription of miR-21 *via* the NF-kB pathway	PTEN repression, increased PI3K/AKT signaling	Up	Up
MALAT1 [104]	HepG2, SMMC-7721	Sponges miR-140-5p	Increased proliferation, migration, and EMT; decreased apoptosis in sorafenib-resistant cells	Up	Up
TUG1 [105]	SMMC-7721, HepG2, BEL-7402	N/A	Increased proliferation, migration, and invasion; decreased apoptosis	Up	Up
KDM4A-AS1 [20]	Hep3B, HuH7 BALB/c nude mice (xenograft)	Recruits ILF3 to stabilize AURKA mRNA	Reduced apoptosis and increased EMT	Up	Up
TIALD [107]	SMMC-7721, SNU-449, SK-Hep-1 B-NDG	Negatively regulated by METTL16; promotes AURKA lysosomal degradation	Increased EMT and metastasis	Up	Down
circHMGCS1 [106]	SMMC-7721	Sponging of miR-581	miR-581 downregulation (predicted), leading to AURKA upregulation	Up	Up

### 4.3. LncRNAs Modulating AURKA Expression in HCC

In exploring the intricate regulatory landscape of AURKA in HCC, it becomes evident that lncRNAs exert diverse regulatory effects on *AURKA* expression through various mechanisms [108,109,110]. Some lncRNAs serve as competitive endogenous RNAs (ceRNAs) by sponging miRNAs that downregulate *AURKA* and thus indirectly regulating *AURKA* expression [111]. Conversely, other lncRNAs modulate AURKA expression through distinct mechanisms independent of miRNA regulation [20,107]. Therefore, categorizing lncRNAs based on their mechanisms provides a comprehensive framework for understanding their roles in *AURKA* dysregulation in HCC. Bioinformatics-based tools have facilitated the high-throughput identification of key dysregulated lncRNAs in HCC as well. For instance, two differentially expressed lncRNAs (*CTD-2510F5.4* and *HAND2-AS1*) were identified along with *AURKA* in HCC samples based on The Cancer Genome Atlas (TCGA) data. Although *CTD-2510F5.4* and *HAND2-AS1* were predicted to modulate and interact with AURKA in HCC based on the integrated analyses, the authors were unable to experimentally validate the expression of these targets in HCC tissues [112].

#### 4.3.1. LncRNAs as Molecular Sponges

Metastasis-associated lung adenocarcinoma transcript-1 (*MALAT1)* is a well-studied lncRNA overexpressed in many solid tumors, including HCC [113,114,115,116]. It promotes cancer progression due to its involvement in metastasis and drug resistance, coupled with its correlation with poor prognosis [117,118]. *MALAT1* levels are significantly elevated in sorafenib-resistant HCC cells (HepG2 and SMMC-7721). When *MALAT1* is overexpressed, it promotes cell proliferation, survival, migration, and expression of mesenchymal markers (N-cadherin and Vimentin) [104]. *MALAT1* induces these functional consequences by sponging miR-140-5p, which was shown to indirectly upregulate *AURKA* in sorafenib-resistant HCC cells [104] (Table 1). The existence of the MALAT1/miR-140-5p/AURKA axis highlights its potential as a therapeutic target in addressing sorafenib resistance in HCC management; indeed, its inhibition enhanced sorafenib efficacy in a nude mice model (Figure 2) [104]. In patients with HCC, *MALAT1* expression showed a negative correlation with miR-140-5p levels and a positive correlation with *AURKA* levels [104].

Another lncRNA regulating the expression of *AURKA* in HCC is Taurine-upregulated gene 1 *(TUG1*). *TUG1* is upregulated in HCC cells (SMMC-7721, HepG2, and BEL-7402) compared to the normal liver cell line, L-02. *In vitro*, the overexpression of *TUG1* by transfecting HCC cells with a *TUG1* RNA-mimic induced proliferation, migration, and invasion while reducing apoptosis in these cells. *TUG1* mimic also increased both mRNA and protein levels of AURKA, suggesting its influence on the malignant phenotype of the mentioned cell models through targeting AURKA (Figure 3 and Table 1) [105]. Although the precise mechanism by which *TUG1* regulates AURKA in HCC was not demonstrated in the study, consistent evidence showed that *TUG1* positively modulates AURKA in adult acute myeloid leukemia (AML). The significantly elevated levels of *TUG1* in adult AML patients (n = 186) positively correlated with poor prognosis and other characteristic features of the disease such as white blood cell count, monosomal karyotype, and FMS-like tyrosine kinase-3 internal tandem duplication (FLT3-ITD) mutation. Lentiviral vector transfection of *TUG1* mimic into the KG-1 AML cell line increased AURKA mRNA and protein expression. The *TUG1* mimic also promoted cell proliferation and survival of AML cells [119]. The pro-tumorigenic functional effects of *TUG1* and its positive regulatory role on AURKA expression are also corroborated by the findings of Li and colleagues in the context of epithelial ovarian cancer (EOC) [120]. However, the exact mechanism that determines the positive regulation of AURKA by *TUG1* still remains unknown both in HCC and other cancers [121,122].

#### 4.3.2. Alternative Mechanisms of lncRNA-Mediated Regulation of AURKA

Besides acting as miRNA sponges, lncRNAs can also modulate *AURKA* by recruiting protein complexes to stabilize its mRNA [123]. The lncRNA *KDM4A antisense RNA 1 (KDM4A-AS1)* was significantly elevated in HCC tissues, especially in stage III+IV HCC patients, as well as in HCC cell lines (Hep3B and HuH7). The knockdown of *KDM4A-AS1* in HCC cells elevated the pro-apoptotic marker BAX2 and concomitantly reduced the expression of BCL2, indicative of an anti-apoptotic effect of this lncRNA [20]. This diminished proliferation and increased E-CADHERIN expression are suggestive of EMT inhibition. Interestingly, RNA pull-down experiments demonstrated a direct interaction between *KDM4A-AS1* and Interleukin enhancer binding factor 3 (ILF3) [20]. Knocking down *KDM4A-AS1* or *ILF3* in Hep3B and HuH7 cells resulted in a time-dependent decline in *AURKA* mRNA, up to 12h, and a significant downregulation of AURKA protein in both cell lines (Table 1) [20]. Thus, this evidence has established the involvement of the *KDM4A-AS1*/ILF3 complex in the regulation of *AURKA* stability and expression in HCC (Figure 3). The authors also demonstrated that E2F transcription factor 1 (E2F1) binds to the promoter region of *KDM4A-AS1* to promote transcription, thus starting the cascade that ultimately results to EMT in HCC cells. This was further explored *in vivo* (BALB/c male nude mice), where the repression of E2F1 decreased tumor volume and weight, whereas the overexpression of *KDM4A-AS1* reversed these parameters. Interestingly, co-transfection of sh-E2F1 and overexpressing (OE)-*KDM4A-AS1* significantly elevated *AURKA* compared to sh-E2F1 mice setups [20]. Thus, we can speculate that the feedback loop between AURKA and E2F1 also exists in HCC, as was previously demonstrated by He and colleagues. They observed that AURKA elevated the transcriptional activity of E2F1; while both proteins were found to be elevated in breast cancer tissues [103].

A recent study by Wang et al. unveiled a novel mechanism by which lncRNAs regulate the lysosomal degradation of AURKA. Transcriptomic analyses of HCC tissues revealed the downregulation of Transcript inducer of AURKA lysosomal degradation (*TIALD*) (Figure 3) [107]. This downregulation was associated with vascular invasion, poor differentiation, recurrence, and low overall survival (OS) of patients [107]. Functional studies confirmed the role of *TIALD* in mediating EMT and metastasis in HCC [107]. The knockdown of *TIALD* in SMMC-7721 cells and overexpression in SNU-449 and SK-Hep-1 cells, as well as in a female B-NDG mice model, demonstrated its significant impact on promoting EMT and metastasis both *in vitro* and *in vivo* [113]. In HCC, *TIALD* is repressed due to N6-methyladenosine (m6A) modifications mediated by m6A methyltransferase (METTL16) [107]. RNA pull-down experiments confirmed METTL16 as a protein interacting with *TIALD*. Indeed, METTL16 knockdown determined an increased TIALD1 expression [107]. On the contrary, the overexpression of the m6A demethylase AlkB homolog 5 RNA demethylase (ALKBH5) significantly enhanced *TIALD* expression, while YTH N6-methyladenosine RNA binding protein 2 (YTHDF2) and YTH domain containing 1 (YTHDC1) accelerated *TIALD* degradation in HCC cells (SMMC-7721 and SNU449) [107]. Additionally, clinical tissue samples and TCGA data indicated a negative correlation between METTL16 expression and *TIALD* levels, further substantiating the involvement of METTL16 in *TIALD* downregulation in HCC. The reduction in METTL16 in HCC contributes to the stability of *TIALD*. Consequently, *TIALD* binds and marks AURKA for lysosomal localization and degradation in HCC cells (SNU449) [107] (Figure 3).

The diverse regulatory mechanisms utilized by lncRNAs in modulating AURKA expression highlight the multifaceted nature of their roles in HCC. Through ceRNA activity, lncRNAs such as *MALAT1* and *TUG1* sponge miRNAs, indirectly regulating AURKA. Additionally, lncRNAs like *KDM4A-AS1* recruit protein complexes to stabilize *AURKA* mRNA, further demonstrating the versatility of lncRNAs in dysregulating AURKA (Table 1). Intriguingly, the mechanism by which TIALD regulates AURKA may provide a novel approach to tackling lncRNAs in HCC.

##### 4.4. circRNAs Regulating AURKA in HCC

The regulatory landscape of circRNAs-AURKA in HCC is less studied compared to miRNAs and lncRNAs, even though accumulating evidence has demonstrated that the dysregulation of circRNAs plays a pivotal role in tumor development and progression [124,125,126]. Similar to lncRNAs, many circRNAs downregulate key tumor-suppressor miRNAs by sponging them, thereby increasing the expression of the miRNA targets [127]. Wang and colleagues explored two circRNA microarray datasets (GSE94508 and GSE97332) and identified circHMGCS1 (hsa_circ_0072389) as significantly dysregulated in HCC tissues compared to paired normal tissues. The upregulated expression of circHMGCS1 was further verified in SMMC-7721 cells (Figure 3). By exploring the expression profiles of HCC circRNAs, miRNAs, and mRNAs in TCGA and the Gene Expression Omnibus (GEO) databases, the authors identified a circRNA-miRNA-hub gene regulatory network, including seven target hub genes (Cbp/p300 interacting transactivator with Glu/Asp rich carboxy-terminal domain 2 [CITED2], Acyl-CoA synthetase long-chain family member 4 [ACSL4], Myristoylated alanine-rich protein kinase C substrate [MARCKS], Kinesin family member 5B [KIF5B], AURKA, Smoothened, frizzled class receptor [SMO], and Ras homolog family member A [RHOA]). Within this network, circHMGCS1 targeted miR-581, which, in turn, can target *AURKA* in HCC, thus revealing the possible existence of a circHMGCSA/miR-581/AURKA regulatory axis [106] (Figure 3). However, further experimental evidence is necessary to prove the the role and functional effects of this predicted regulatory axis in HCC.

## 5. Clinical Implications and Future Directions

The intricate interplay between ncRNAs and AURKA in HCC offers profound clinical implications, shaping diagnostic, prognostic, and therapeutic paradigms in cancer management. However, their full clinical utility remains challenging in terms of their diagnostic and prognostic significance as well as their potential as candidate targets for drug development. 

### 5.1. Diagnostic and Prognostic Significance

The regulatory network involving ncRNAs and AURKA is a promising source of diagnostic and prognostic biomarkers in HCC and other cancers. Dysregulated expression profiles of various ncRNAs, particularly miRNAs, lncRNAs, and circRNAs, intricately modulate AURKA expression and activity. This impacts pivotal oncogenic processes such as proliferation, metastasis, and resistance to therapy [106,107,128]. For instance, tumor-suppressor miRNAs such as and miR-199b-3p [96], miR-129-3p [99], miR-490-3p [89], and miR-26a-5p [101] directly repress *AURKA*, whereas lncRNAs like *MALAT1* [104] and *TUG1* [120] indirectly upregulate *AURKA* by sponging and downregulating tumor-suppressor miRNAs.

Elevated levels of lncRNAs such as *MALAT1* and *TUG1*, coupled with reduced expression of miRNAs like miR-129-3p, correlated significantly with poor prognosis in HCC patients [97,104,116,120,129,130]. Therefore, aberrant ncRNA signatures may serve as robust biomarkers for predicting tumor aggressiveness, metastatic potential, and patient survival and potential recurrence. Serum miR-21 demonstrated promising diagnostic value in an Egyptian cohort, showing high specificity and sensitivity as a non-invasive biomarker for HCC [131]. miR-21 is particularly interesting, since high AURKA levels indirectly increased its transcription, resulting in the downregulation of the key tumor suppressor, PTEN [24], which was reported to confer resistance to therapy in HCC [132]. Importantly, the differential expression patterns of ncRNAs between HCC tissues and adjacent, non-tumoral tissues provide a foundation for developing non-invasive diagnostic tools and prognostic markers, facilitating early disease detection and tailored therapeutic interventions.

However, the clinical utility of these ncRNAs requires extensive validation in the clinical setting prior to being categorized as *bona fide* diagnostic and prognostic markers or may be in the form of signatures/profiles in cancer.

### 5.2. Therapeutic Targeting

Systemic therapies such as sorafenib [133,134] and lenvatinib [135,136] have been the mainstay treatment options, especially for advanced HCC. These drugs target pathways, particularly those involved in angiogenesis and cell proliferation. Despite their broad mechanism of action, their efficacy is frequently undermined by the development of therapeutic resistance and significant adverse effects [133,137]. More recently, immune checkpoint inhibitors like nivolumab [138] and pembrolizumab [139] demonstrated promise in advanced cases as well by boosting anti-tumor immune responses. However, these treatments only benefit a subset of patients, underscoring the need for more targeted treatment options. This underscores the pressing need to expand the already narrow range of therapeutic options and to develop a more refined, targeted therapeutic approach that can address the heterogeneous molecular drivers of HCC.

One such strategy involves exploiting the dysregulated ncRNA-AURKA axis, which has emerged as a compelling therapeutic strategy. By modulating the expression or activity of key ncRNAs implicated in AURKA regulation, therapeutic interventions can effectively impede tumor development and progression and therefore improve prognosis. For instance, restoring the expression of tumor-suppressive miRNAs or suppressing oncogenic lncRNAs can attenuate AURKA expression, thereby inhibiting cancer cell proliferation, migration, and invasion as well as reducing the risk of resistance to standard anti-tumor agents [80,89,107,110,128]. Nevertheless, the downsides of targeting AURKA, in a highly heterogeneous cancer such as HCC, cannot be discounted, since not all tumors may be driven by AURKA dysregulation [140]. Like existing targeted drugs on the market, prolonged AURKA targeting may also result in drug resistance. Furthermore, off-target effects also limit the efficacy of AURKA as a monotherapy, given that AURKA also plays a key role in cell division in healthy cells, which may lead to negative side effects among patients [141].

Although several pre-clinical studies have presented evidence about the potential benefits of targeting lncRNAs and circRNAs, clinical trials investigating these ncRNAs have not commenced yet. For instance, *MALAT1* is a promising target using anti-sense oligonucleotides (ASO) in breast [142] and lung [143] cancers. Given its upregulated expression in HCC, *MALAT1* may prove to be a viable target, potentially with ASOs, due to its clear involvement in metastasis [117,144,145], which remains an unmet clinical need in HCC management [146,147]. Furthermore, the development of small molecules or peptide inhibitors targeting interactions between lncRNAs and their miRNA targets represents a novel avenue for therapeutic development as well. Hence, delineating a particular ceRNA network in HCC is imperative for this kind of approach.

## 6. Conclusions

The intricate interplay between ncRNA and AURKA in HCC underscores their pivotal roles in tumorigenesis, disease progression, and therapy resistance. Tumor-suppressive miRNAs directly target and repress *AURKA*, while oncogenic lncRNAs and circRNAs enhance *AURKA* levels primarily by sponging tumor-suppressor miRNAs, thereby driving oncogenic processes. Elucidating these mechanisms has revealed novel aspects of AURKA regulation that warrant further investigation. Notably, AURKA has a pseudogene, *AURKAPS1*, which acts as a miRNA sponge and is overexpressed in HCC, adding an intriguing layer to our understanding of the regulatory landscape of AURKA and ncRNAs in HCC [148].

Importantly, these findings hold substantial clinical implications, with the potential to benefit multiple facets of HCC management. The dysregulation of ncRNAs in cancer represents a promising opportunity for their use as non-invasive diagnostic and prognostic biomarkers in HCC, especially with the consideration that certain ncRNAs can be detectable in biological fluids. This would be particularly beneficial for patients requiring regular surveillance, improving detection rates in high-risk populations and facilitating timely therapeutic intervention.

From a therapeutic perspective, targeting ncRNA-AURKA interactions represents a novel approach in overcoming therapeutic resistance, which remains a major hurdle in HCC treatment. Restoring tumor-suppressive miRNAs or inhibiting oncogenic lncRNAs and circRNAs may offer a promising avenue for reducing cancer cell survival, proliferation, and migration. This could enhance the efficacy of already available drugs, including chemotherapy, targeted drugs, and even immunotherapy.

Future research should prioritize the comprehensive profiling of ncRNA and AURKA expression in large HCC patient cohorts and the investigation of their context-dependent roles. Additionally, exploring the synergistic potential of AURKA-targeted therapies in combination with ncRNA modulation and existing treatment modalities could lead to more personalized and effective therapeutic strategies. This integrated approach holds the promise of advancing HCC detection, management, treatment, and prognoses for patients.

## Data Availability

No new data were created or analyzed in this study. Data sharing is not applicable to this article.
